# Suppression of *Fusarium* Wilt Caused by *Fusarium oxysporum* f. sp. *lactucae* and Growth Promotion on Lettuce Using Bacterial Isolates

**DOI:** 10.4014/jmb.2104.04026

**Published:** 2021-08-04

**Authors:** Dil Raj Yadav, Mahesh Adhikari, Sang Woo Kim, Hyun Seung Kim, Youn Su Lee

**Affiliations:** Department of Applied Plant Sciences, Interdisciplinary Program in Smart Agriculture, Kangwon National University, Chuncheon 24341, Republic of Korea

**Keywords:** Antifungal activity, biocontrol, *Fusarium oxysporum* f. sp. *lactucae*, rhizobacteria

## Abstract

This study was carried out to explore a non-chemical strategy for enhancing productivity by employing some antagonistic rhizobacteria. One hundred eighteen bacterial isolates were obtained from the rhizospheric zone of various crop fields of Gangwon-do, Korea, and screened for antifungal activity against Fusarium wilt (*Fusarium oxysporum* f. sp. *lactucae*) in lettuce crop under in vitro and in vivo conditions. In broth-based dual culture assay, fourteen bacterial isolates showed significant inhibition of mycelial growth of *F. oxysporium* f. sp. *lactucae*. All of the antagonistic isolates were further characterized for the antagonistic traits under in vitro conditions. The isolates were identified on the basis of biochemical characteristics and confirmed at their species level by 16S rRNA gene sequencing analysis. *Arthrobacter sulfonivorans*, *Bacillus siamensis*, *Bacillus amyloliquefaciens*, *Pseudomonas proteolytica*, four *Paenibacillus peoriae* strains, and *Bacillus subtilis* were identified from the biochemical characterization and 16S rRNA gene sequencing analysis. The isolates EN21 and EN23 showed significant decrease in disease severity on lettuce compared to infected control and other bacterial treatments under greenhouse conditions. Two bacterial isolates, EN4 and EN21, were evaluated to assess their disease reduction and growth promotion in lettuce in field conditions. The consortium of EN4 and EN21 showed significant enhancement of growth on lettuce by suppressing disease caused by *F. oxysporum* f. sp. *lactucae* respectively. This study clearly indicates that the promising isolates, EN4 (*P. proteolytica*) and EN21 (*Bacillus siamensis*), can be commercialized and used as biofertilizer and/or biopesticide for sustainable crop production.

## Introduction

Naturally occurring bacteria have been suggested as a replacement for supplements and chemical pesticides to control plant diseases [1]. Various species of bacteria have been focused on because of their root-colonizing capacity as well as their catabolic adaptability and production of metabolites with antibacterial and antifungal efficacy [2]. Several species of soil and seed-borne plant pathogenic fungi, such as *Fusarium*, *Sclerotinia*, *Colletotrichum*, *Rhizoctonia* and *Pythium*, are distributed globally and are known to cause significant economic losses to food and vegetable crop yields. *Fusarium* is one of the most important genera of plant pathogenic fungi with a record of devastating infections in various economically important plants [2, 3].

Control of all these phytopathogens is hugely based on genetic resistance in the host plant, use of synthetic pesticides, and environmental factors as well as management of the plant pesticides [4].

However, the use of chemical pesticides leads to inequality in the microbial community and may create new strains of resistant pathogens against beneficial microorganisms [5]. Therefore, the beneficial effects of rhizobacteria towards various phytopathogens can be explored for sustainable crop production [6, 7].

Soil bacteria and fungi possess some vital processes such as nitrogen fixation, nutrient mineralization and mobilization, decomposition and denitrification. The motility of bacteria has great impact on their ability to thrive in soil and colonization in the beginning phases where movement and attachment to the root surface are crucial [8]. Hence, essential identification of bacteria mostly involves the determination of colony morphology, catalase and oxidase testing, Gram staining, Voges-Proskauer tests (IMViC), and utilization of sugars (IMViC) [9].

Characterization of bacterial isolates via biochemical assay using organic manure and solid waste degradation was carried out based on IMViC and catalase oxidase through testing [10].

Applying environment friendly biocontrol agents is a specific and natural way to control plant pathogens and increase crop production [11].Several studies have been reported regarding the suppression of plant pathogens under in vitro and in vivo conditions using *Paenibacillus* and *Bacillus* species [12]. Production of vegetable crops including lettuce, watermelon and tomato has been decreasing due to infection caused by *F. oxysporum* f. sp. *lycopersici*, *F. oxysporum* f. sp. *lactucae*, and *F. oxysporum* f. sp. *melonis*, respectively, and these pathogens now pose a major threat to commercial vegetable growers around the world. Ample studies have reported that rhizobacterial isolates showed a significant role in suppressing notorious plant pathogens like *F. oxysporum*, *R. solani*, *P. infestans* [13, 14], *Serratia marcescens* [15], *Gliocladium roseum*, *Penicillium* sp. [16], and *Pythium radiosum* [17].

The main objectives this study were to (i) isolate the potential antagonistic rhizobacteria from various sources and (ii) suppress Fusarium wilt caused by *Fusarium oxysporum* f. sp. *lactucae* in lettuce crop under in vitro and in vivo conditions.

## Materials and Methods

### Soil Sample Collection and Isolation of Bacterial Isolates

In total, 25 soil samples were collected from Chuncheon (37°56'21.69'' N, 127°46'55.30'' E), Hongcheon (37°41'46.74'' N, 127°54'19.01'' E), and Hwacheon (38°06'37.99'' N, 127°41'14.02'' E) in Gangwon-do, Korea, during April to June 2014. Soil sample collection was performed following the method of Harley and Waid (1975)[18]. Soil samples were dug about 2-3 inches from the immediate vicinity of rice (*Oryza sativa* L.), maize (*Zea mays* L.), soybean (*Glycine max* (L.) Merr.), oat (*Avena sativa* L.), and sesame (*Sesamum indicum* L.) roots. Soil was collected by withering the roots into polythene bags. Collected soil was sieved using an autoclave-sterilized brass sieve of 2 mm aperture size. Soil samples were stored at 4°C in Ziploc polythene bags for further use. Ten oat plants were randomly collected from an oat field of an experimental farm of Kangwon National University, Gangwon-do, Korea, and transported on ice to the laboratory for isolation of endophytic bacteria. Roots were excised and cleaned under running tap water to remove any adhering soil and then air dried and processed within 5 h of collection. Roots were cut into segments of 5-10 mm long. Root segments were surface sterilized by immersion in 70% ethanol for 3 min, washed with fresh sodium hypochlorite solution (2.5% available chlorine) for 5 min, rinsed with 70% ethanol for 30 s, and finally washed five times with sterile distilled water to remove the sterilization agents. After the treatment, root tissues were soaked in 10% NaHCO3 solution to suppress the growth of endophytic fungi. Soil dilutions were prepared with one gram of soil sample suspended in 9 ml of sterile distilled water and vigorously shaken to mix properly for 2 min. Rhizospheric bacteria were isolated from serially diluted soil sample solutions. Dilution of 10-5 and 10-6 from 1 ml soil solution was plated in Petri plates containing TSA (Tryptic Soy Agar, Difco Laboratories, USA) medium [19]. For the isolation of endophytic bacteria, five root pieces from each sample were placed into TSA Petri plates and incubated for 2-3 days at 28°C. Bacterial colonies of different morphological appearance were picked and re-cultured on a fresh Petri plate into TSA medium in order to obtain pure colonies. Stock cultures of each bacterial isolate were prepared in TSB (Tryptic Soy Broth) containing 20% glycerol and kept at -80°C for further use.

### Fungi and Culture Conditions

The fungal pathogen (*Fusarium oxysporum* f. sp. *lactucae*) used in this study was obtained from the Korean Agricultural Culture Collection (KACC, No. 40032). The pathogenic fungi were cultured on potato dextrose agar (PDA, Difco) supplemented with 100 μg/l (bacteriostatic agent) and incubated for 7-10 days at 28°C. The freshly prepared pure cultures of fungal pathogen on the PDA plugs were stored at 4°C for further use.

### Screening of Bacteria Antagonistic to *Fusarium oxysporum* f. sp. *lactucae*

All bacterial isolates were screened for antagonistic activity against tested fungal plant pathogens using dual plate culture technique [20] with slight modification. Briefly, 6 mm mycelial plugs of actively growing pathogens were placed on the center of plates (150 mm diameter) containing PDA medium, and then eight sterilized paper discs (6 mm diameter) were placed equidistantly about 1.5 cm from the edge of the same plate. Suspensions of 10 μl of each bacterial isolate were inoculated in the paper discs and incubated for 7 days at 28°C until the fungus in the control plate covered the edge of the plate. The plates without bacterial inoculation and containing fungal plugs only were considered as control. Scoring technique was applied to measure the mycelial growth inhibition of pathogen. Zero (0) indicates the bacterial isolates fully covered with hyphae of fungi, one (1) indicates the fungal hyphae at the edge of the bacterial colony and, two (2) indicates a clear fungal mycelial inhibition zone around the bacterial colony [21]. The isolates with a score of 2 were only considered as antagonists and were selected for further evaluation of antagonistic properties by different methods.

### Determination of Percentage Inhibition of Mycelial Growth of Fungal Pathogens

Antagonistic efficacy of screened bacterial isolates was further evaluated by dual culture technique. Briefly, 6 mm mycelial plugs of actively growing pathogens were placed on the center of Petri dishes (90 mm) containing 25 ml PDA-TSA (1:1 v/v), and then three sterilized paper discs (6 mm) were placed equidistantly about 1.5 cm from the edge of the same plate. Each paper disc was inoculated with a 10 μl freshly grown bacterial suspension at the concentration of 10^8^ CFU/ml. The plates without bacterial inoculation and containing fungal plug only were considered as control. The test was done in triplicate. The antagonistic effect was determined by measuring the size of the inhibition zones and the radial growth of fungal mycelium. The percent inhibition of growth over control was calculated using this formula:

$$\text{\% inhibition = }\left[1-\left(\frac{\text{Fungal growth in treatment}}{\text{Fungal growth in control}}\right)\right]\times 100.$$

### Inhibition of Fungal Mycelium Proliferation by Broth Culture Assay

One milliliter of 48-hour-old bacterial culture and two discs of 6 mm of test fungi were inoculated in 50 ml of PDB and TSB (1:1 v/v) in a conical flask of 250 ml at 28°C on a rotary shaker at 150 rpm (replications were made thrice per isolate). Control represents the broth inoculated with fungus only. The differences in dry weights between the bacterium treated and the control cultures were recorded by passing dual cultures grown for 7 days through pre-weighed filter paper. The filter papers were dried for 24 h at 70°C and weighed. The experiment had a completely randomized design with three replications. The reduction in weight of the test fungi was calculated using this formula in percentage [22]:

Reduction in weight (%) = (W1-W2)/W1×100,

where, W1 represents the weight of the test fungus in control flasks and W2 with the bacterial antagonists.

### Elucidation of Antagonistic Traits

**Chitinase activity.** Qualitative estimation of chitinase was carried out in chitin agar plates prepared and amended with 2% phenol red and isolates (10 μl) were inoculated into wells. The plates were incubated for 120 h at 25-29°C and the chitinase activity was indicated as clear halos around the inoculated holes. The magnitude of the activity was calculated by measuring the diameter of the zones. The test was repeated in triplicate for each isolate [23].

**Protein hydrolysis.** Skim milk agar plates (skim milk 100 g, peptone 5 g, agar 15 g, distilled water 1,000 ml) were prepared and inoculated with pure bacterial culture into wells. The inoculated plates were incubated at 28°C for 48 h, and the plates were observed for clear zones around the wells [24].

**Pectinase and cellulase production.** To determine pectinase and cellulose production, the media were prepared by adding 1% pectin and cellulose in basal medium (NaNO3 1 g, K2HPO4 1 g, KCl 1 g, MgSO4.7H2O 0.5 g, yeast extract 0.5 g, glucose 1 g, distilled water 1,000 ml, agar 15 g). Ten microliters of the bacterial cell suspension was inoculated into the wells made on the medium and incubated for 5 days at 28°C. Gram’s iodine solution (3%) was poured in the pectin and cellulose agar media and zones of clearance were observed against the dark blue background. A clear zone against the blue background indicated that the bacteria were positive for pectinase and cellulase production. The magnitude of the activity was calculated by measuring the diameter of the zones. The test was repeated in triplicate for each isolate [24].

### Elucidation of Plant Growth-Promoting Traits

**Hydrogen cyanide (HCN) production.** Nutrient agar amended with 4.4 g/l glycine and bacteria was streaked (log phase) onto plates. A Whatman filter paper No. 1 soaked in 2% sodium carbonate in 0.5% picric acid solution was placed at the top of the plates which were then sealed with parafilm and incubated at 35-37°C for 4days. Development of orange to red color indicated HCN production [25].

**Hydrolysis of starch.** Starch agar plates (peptone 5 g, beef extract 3 g, soluble starch 0 g, agar 15 g, distilled water 1,000 ml) were prepared and inoculated with pure bacterial culture and incubated at 25-29°C for 48 h. After incubation, iodine (3%) was poured onto the plates. Formation of a blue-black color due to starch-iodine complex in the unutilized places of starch in the agar plates was indicated. Starch hydrolysis by the bacteria via production of amylase was indicated by a clear halo zone surrounding the bacterial colony on the starch agar medium. The test was repeated thrice for each culture and recorded [24].

**Siderophore production.** Siderophore production by bacterial isolates was detected by the universal method of Schwyn and Neilands (1987) [26] using chrome azurol S (CAS) media. CAS agar plates were prepared and inoculated with the 10 μl of exponentially growing test bacterial culture (0.5 OD at 620 nm) and incubated at 28°C for 7 days. Development of a yellow-orange halo around the colony was considered as positive for siderophore production. The test was repeated thrice for all the cultures and siderophore production efficiency (SPE) was calculated by the following formula:

$$\text{SPE = }\frac{\text{Colony diameter (mm)+Diameter of the halo zone (mm)}}{\text{Colony diameter (mm)}}$$

**Ammonia production.** Bacterial isolates (50 μl of bacterial cell suspension) were grown in 30 ml peptone water broth (4%) for five days at 25-29°C. Two milliliters of culture supernatant was mixed with 1 ml Nessler’s reagent and a volume of this mixture was increased to 8.5 ml by addition of ammonia-free distilled water. Development of yellow-to-brown color indicated ammonia production, and the optical density was measured at 450 nm using a spectrophotometer. The concentration of ammonia was estimated using the standard curve of ammonium sulphate in the range of 0.1-1.0 μmole/ml.

**Indole acetic acid production (IAA).** IAA production was estimated using the method described by Bric *et al*., 1991 [27].

Ten percent exponentially grown bacterial strain culture was inoculated in 100 ml NB (or 50 μl cell suspension in 5 ml of the sterile peptone yeast extract broth (peptone 10 g, beef extract 3 g, NaCl 5 g), with varying concentrations of L-tryptophan ranging from 0 to 500 μg/ml in a 15-ml tube. The broth (2 ml) was collected at 24, 48, and 72 h and centrifuged at 2,700 g for 15 min followed by assay for quantitative measurement of IAA. Then, 1 ml of the cell-free supernatant was mixed vigorously with 1 ml Salkowsky’s reagent (1 ml of 0.5M FeCl_3_ in 50 ml of 35% HClO_4_-perchloric acid) along with two drops of orthophosphoric acid and the assay system was kept at room temperature (25-29°C) in dark for 20 min till pink color developed (in a 2-ml Eppendorf tube). Optical density was measured spectrophotometrically at 535 nm. The concentration of IAA in each sample was determined from the standard curve of IAA with the standards prepared in the range of 10-100 μg/ml of IAA [28].

**Phosphate solubilization.** Phosphate solubilization activity of the selected rhizobacterial isolates was detected by means of plate assay using Pikovskaya (PVK) agar, which results in a clear halo formation. A pure colony from a fresh culture of each isolate was inoculated at four equidistant points into each of the PVK-agar media using a sterile needle. The diameter of the clear halo zone was observed after 12 days of incubation at 28°C. Control plates were inoculated with sterile tryptic soy broth (TSB) only. The diameters of the colony and clearing zones around the colonies were measured. All the tests were replicated thrice. The solubilization index of the isolates was calculated with the formula given below:

$$\text{Solubilization index (SI)}=\frac{\text{Colony diameter (mm)+Diameter of the halo zone (mm)}}{\text{Colony diameter (mm)}}$$

**Zinc solubilization.** The selected antagonistic bacterial isolates were inoculated into modified PVK medium (ingredients g/l), (glucose 10.0 g, ammonium sulphate 1.0 g, potassium choloride 0.2 g, dipotassium hydrogen phosphate 0.2 g, magnesium suphate 0.1 g, yeast 0.2 g, distilled water 1,000 ml, pH 7.0) containing 0.1% insoluble zinc compounds ( ZnO, ZnCO_3_, and ZnS). The test organisms were inoculated on these media and incubated at 28°C for 7 days. The diameters of the clear zone around the colonies were measured. All the tests were replicated thrice. The solubilization index of the isolates was calculated with the formula given below:

$$\text{Solubilization index (SI)}=\frac{\text{Colony diameter (mm)+Diameter of the halo zone (mm)}}{\text{Colony diameter (mm)}}$$

**Molecular identification and phylogenetic analysis.** For the extraction of DNA, the bacterial cells were harvested from 10 ml overnight culture and pellets were lysed in 1 ml lysis buffer (25% sucrose, 20 mM EDTA, 50 mM Tris-HCl and 5 mg/ml^-1^ of lysozyme). Chromosomal DNA was extracted following the standard procedure [29]. Universal primers 27F and 1492R were used to amplify the 16 rRNA using PCR [30]. The PCR was carried out in a thermocycler using 35 amplification cycles at 94°C (45 sec), 55°C (60 sec), and 72°C (60 sec) with a final extension for 7 min at 72°C. Products obtained from the PCR were purified by using a Montage PCR Clean-Up Kit (Millipore, USA). Universal primers, 518F and 800R (Macrogen, Korea) were used to sequence the purified PCR products of approximately 1,400 bp through a big Dye Terminator Cycle Sequencing Kit v.3.1 (Applied BioSystems, USA). An Applied BioSystems model 3730XL automated DNA sequencing system (Applied BioSystems) at Macrogen Inc. Seoul, Korea was used to resolved the sequencing products. The sequences were compared using the NCBI (National Center for Biotechnology Information) BLAST (Basic Local Alignment Search Tool) program (http://www.ncbi.nlm.nih.gov/Blast) for identification of the isolates. All positions containing gaps and missing data were eliminated from the dataset. Best hit sequences were downloaded in FASTA format from the NCBI database to construct a phylogenetic tree using MEGA 6 software [31].

**Disease suppression by rapid radicle assay.** The bacterial isolates were cultured in TSB (tryptic soy broth) with shaking at 150 rpm at 28°C for 48 h for bacterial suspensions. Seeds of lettuce were surface sterilized with 5%sodium hypochlorite for 20 min, washed thrice with sterile distilled water and kept in Petri dishes with moist filter paper for 3-4 days at 25°C in darkness for germination. Uniformly germinated seeds were soaked in the bacterial suspensions (10^8^ cells/ml^-1^) of isolates. The treated seeds of lettuce were placed on the margins of actively growing mycelia of *F. oxysporum* f. sp. *lactucae*, grown on water agar amended with 0.02% glucose at 28°C for 5-7 days. These treated plates were incubated at 28°C under 16 h fluorescent light per day until disease expression. Seeds treated with sterile water were served as untreated controls. Disease incidence was evaluated when over 90% of the radicles in untreated controls were infected by tested pathogen. The experiment was laid out in an RCB design with three replications. The number of seeds per replication was ten.

### Greenhouse and Field Evaluations

**Preparation of fungal pathogen inoculum and inoculation technique**. The pure culture of targeted fungal pathogen, *F. oxysporum* f. sp. *lactucae*, was obtained from KACC. The obtained fungal isolates of *Fusarium* spp. were grown on PDA plates at 28°C for 7 days, then in PDB for 14 days at 25°C in a rotary shaker at 150 rpm. After incubation, the conidial suspension was diluted in SDW to give a final concentration of 10^6^ /ml. For each seedling, 50 ml inoculant of tested *Fusarium* pathogen was added to the root zone of three-week-old seedlings of lettuce by pouring the suspension into holes made around the root zone with a sterilized glass rod. The inoculation was done according to the method described by Oh *et al*. (1999) [32] with slight modifications.

**Experimental design and treatments.** The greenhouse and field experiments were set up in RCB (Randomized Complete Block) designs with five replications. Ten bacterial isolates along with positive and negative controls were evaluated for their growth promotion and disease suppression activities on lettuce under greenhouse conditions. For growth promotion experiments, the positive control was maintained by mixing the autoclaved soil with chemical fertilizer (18 N: 7 P: 9 K) of 1 kg/1,000 m^2^ and uninoculated soil was treated as negative control. Three controls, infected with pathogen, non-infected or healthy, and positive (sprayed with 0.2% solution of Mancozeb 75% WP twice at intervals of seven days) were used in the disease evaluation experiments. Two potential bacterial isolates were tested under field conditions for their growth promotion and disease suppression activities on lettuce.

**Observations.** Disease severity (S) for Fusarium wilt of lettuce was estimated (after 5 and 8 weeks of transplanting, respectively), as a wilting percent using the rating scale in which infected plants were classified according to numerical grades ranging from 0 to 4 as follows: 0 = healthy, 1 = ≤ 25% of plant leaflets are yellow and of vascular root bundles are dark brown, 2 = ≥ 26-50% of plant leaflets are yellow and of vascular root bundles are dark brown, 3 = ≥ 50-75% of plant leaflets are yellow and of vascular root bundles are dark brown and 4 = ≥ 76-100% of plant leaflets are yellow and of vascular root bundles are dark brown.

$$\text{DS\% = }\sum \frac{\text{1A+2B+3C+4D}}{4\text{T}}\times 100,$$

where, A, B, C, and D are the number of plants corresponding to the numerical grades 1, 2, 3, and 4, respectively, and 4T is the total number of plants (T) multiplied by the maximum discoloration grade 4, where T = A + B + C + D. Reduction percentage was calculated using the formula of Guo *et al*. (2004) [33] as follows:

$$\text{Reduction \%}=\frac{\text{(disease incidence of control-disease incidence of treatment group)}}{\text{disease incidence of control}}\times 100.$$

**Statistical analysis.** One-way analysis of variance (ANOVA) was applied to analyze the data from in vitro and to determine the significance of treatment effects. The percent data and data set having value zero (0) were transferred into arcsine square root transformation before further statistical analysis to improve the homogeneity of the variance of the data. Where the F values were significant, post hoc comparisons of means were made using Duncan’s multiple range test (DMRT) at the 0.05 probability level. All statistical analyses were done using CROPSTAT version 7.2.3 [34].

## Results

### Culturable Bacteria in the Rhizosphere and Endosphere

Bacteria were obtained both from the rhizospheric portion of various crop plants as well as the root interior of oat plants. Ninety-five bacteria were isolated from rice, maize, barley, sesame and soybean rhizospheric soil; and 23 were recovered from oat root interiors (Table S1). The general isolation frequency was 3.37. The isolation frequency in rhizospheric soil samples of rice, sesame, soybean, maize and oat was 4.86, 4.33, 4.00, 4.00, and 2.50, respectively. The lowest isolation frequency (2.30) was recorded in oat root samples and the highest number of isolates was recorded from rice rhizospheric soil samples.

### Screening of Antagonistic Bacteria

Out of the 118 isolates tested, 20 isolates showed antagonism against all the test pathogens. The number of isolates with a score of 2 was 14 against *F. oxysporum* f. sp. *lactucae*. The isolates that were capable of antagonizing the test pathogen by inducing an inhibition zone around the bacterial colony and having an antagonistic score of 2 were further characterized.

### In Vitro Inhibition of *F. oxysporum* f. sp. Lactucae by Bacterial Isolates

**Fig. 1:**
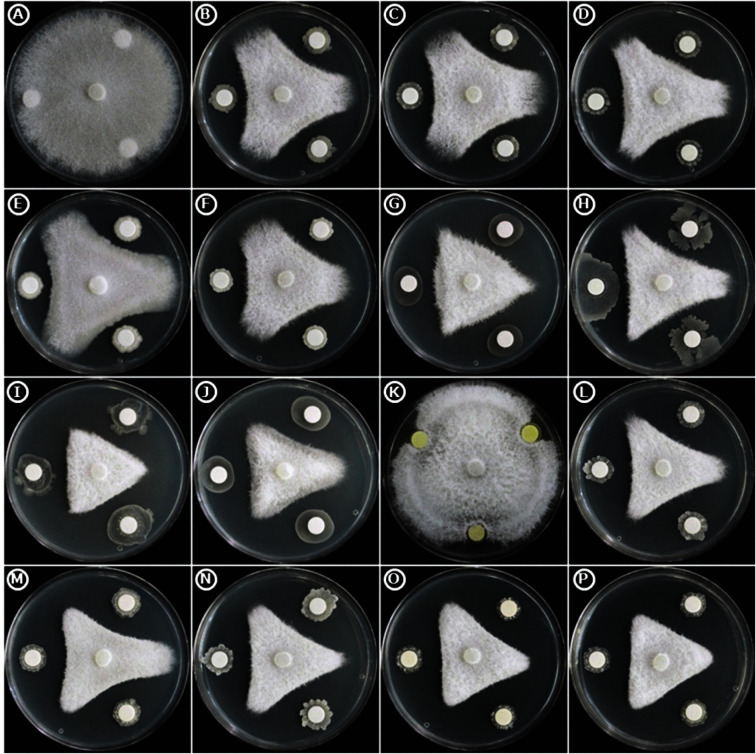
Growth promotion of *Fusarium oxysporum* f. sp. *lactucae* by selected rhizobacterial isolates in dual culture assay. (**A**) Control; (**B**) RR8; (**C**) RR12; (**D**) RR26; (**E**) RR33; (**F**) RR34; (**G**) MR3; (**H**) MR19; (**I**) OR7; (**J**) OR19; (**K**) EN4; (**L**) EN18; (**M**) EN20; (**N**) EN21; (**O**) EN22 and (**P**) EN23.

**Fig. 2:**
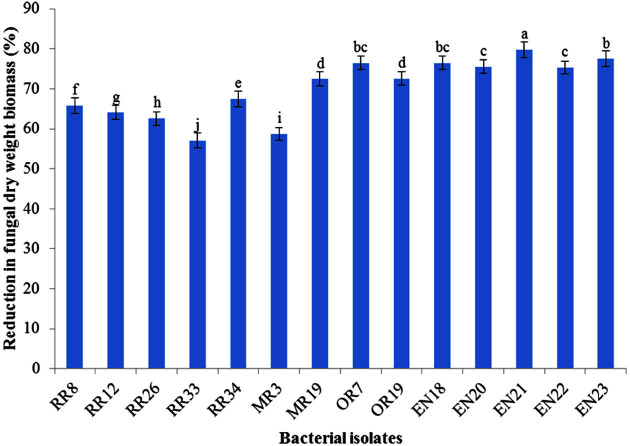
Reduction in mycelial dry weight biomass of *Fusarium oxysporum* f. sp. *lactucae* due to antagonism of rhizobacterial isolates. Values with different lowercase letters indicate significant differences at *p* ≤ 0.05. Error bars indicate the standard error of three replicates.

All the screened bacterial isolates possessed inhibition against the tested pathogenic fungi. The highest inhibition was recorded by EN21 and OR7 (Table 1 and Fig. 1). Moreover, all tested bacterial isolates showed biomass reduction in all tested fungi with varied rate of reduction. The mycelial biomass of all tested fungi was reduced to the highest degree in dual culture broths inoculated with bacterial isolate EN21 (Fig. 2).

### Elucidation of Antagonistic Traits

Fifteen bacterial isolates were tested for antagonistic traits viz., chitinase, protease, pectinase and cellulase production. Clearing of plates containing colloidal chitin as a sole carbon source by the bacterium around the colony was used to measure chitin hydrolysis. All isolates, except RR33 and EN4, showed strong chitinolytic activity (Table 2, Fig. S1). The isolates RR34 and EN4 were weak producers of chitinase. Starch hydrolysis was observed via zones of starch hydrolysis through the production of α-amylase. Clearing of starch agar plates containing starch as a sole source of carbon by the bacterium around the colony was used to measure starch hydrolysis. Out of 15 isolates, 13 isolates were producers of α-amylase. The isolates RR34 and EN4 demonstrated negative response to starch hydrolysis (Table 2, Fig. S2). Clearing of skim milk agar plates containing skim milk as a sole source of protein by the bacterium around the colony was used for qualitative detection of protease production. Out of 15 isolates, 14 isolates demonstrated positive response to protein hydrolysis. The isolate RR34 was found negative with regard to production of protease (Table 2, Fig. S3). Cellulose degradation was observed via zones of cellulose hydrolysis through the production of cellulase. Clearing of agar plates containing cellulose powder as a sole source of cellulose by the bacterium around the colony was used. Out of 15 isolates, 14 isolates demonstrated positive response to cellulose degradation. The isolate EN4 was found negative for the production of cellulase (Table 2, Fig. S4).

### Growth-Promoting Trait Elucidation of Plant

The formation of yellow-to-orange halos was indicative of siderophore production. All tested isolates, except RR8, were positive for siderophore production (Table 2, Fig. S5).

**Fig. 3:**
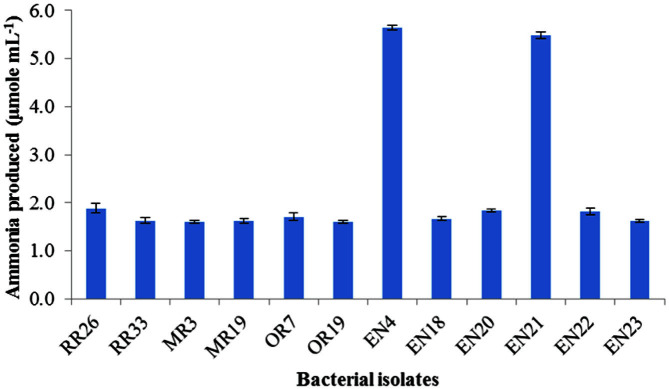
Ammonia production by bacterial isolates. (**A**) Control; (**B**) RR8; (**C**) RR12; (**D**) RR26; (**E**) RR33; (**F**) MR3; (**G**) MR19; (**H**) OR7; (**I**) OR19; (**J**) EN4; (**K**) EN18; (**L**) EN20; (**M**) EN21; (**O**) EN23 and (**P**) RR34.

Bacterial isolates were grown in peptone water broth for detection of ammonia production. Tubes showing faint yellow indicated a small amount of ammonia production, and deep yellow to brownish color indicated a maximum amount of ammonia production. Out of 15 isolates, 12 isolates were positive for ammonia production (Table 3). The isolates RR8, RR12 and RR33 showed negative response to ammonia production. The production of ammonia by the isolates EN4 and EN21 was more evident than the other isolates (Table 3). Maximum ammonia produced by the isolates EN4 and EN21 was 5.7 and 5.6 μmole/ml, respectively (Fig. 3 and Table 3).

**Fig. 4:**
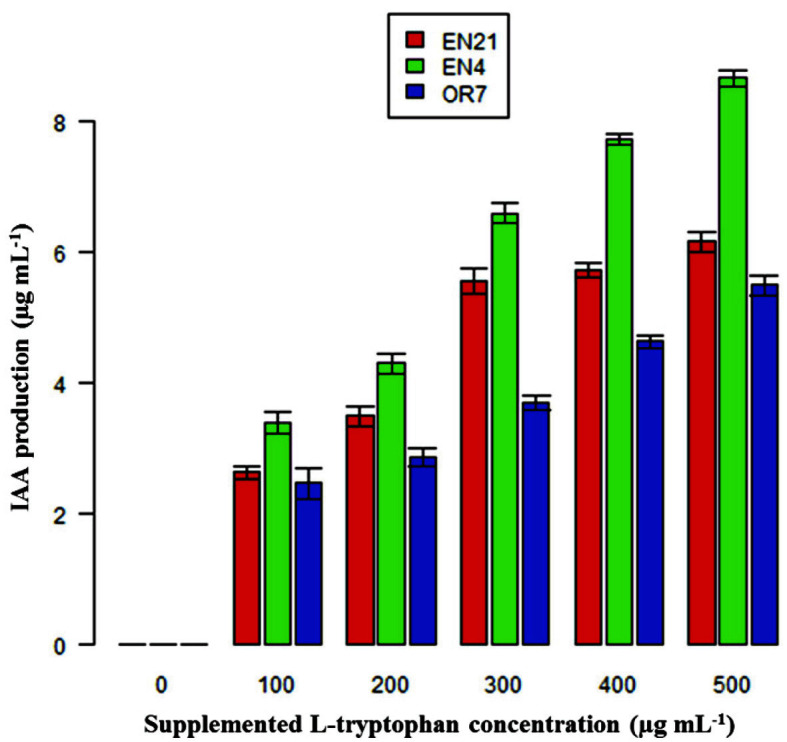
Indole acetic acid produced by selected bacterial isolates at 72 h of incubation in different concentrations of L-tryptophan supplemented in nitrogen free broth. Error bar denotes the standard error of three replicates.

It was observed that out of 15 isolates, only three isolates OR7, EN4 and EN21 could produce IAA only when L-tryptophan was supplemented in the medium. IAA production by the isolates was determined after 72 h of incubation and maximum IAA produced was 8.6 μg/ml by the isolate EN4 when L-tryptophan concentration in the medium was maximum (500 μg/ml) (Fig. 4). In the growth medium with absence of L-tryptophan, IAA was not detected in any of the three isolates even after 72 h (Fig. 4 and Table 3). This shows that there is a direct correlation between IAA production and supplemented L-tryptophan in the medium.

The bacterial isolates that showed zones of clearance on PVK agar media were considered as phosphate solubilizers and the phosphate solubilization index of all 15 bacterial isolates is shown in Table 3. Out of 15 isolates, five isolates demonstrated phosphate solubilization activity. The isolates RR8, RR12 and EN23 showed low solubilization efficiency while the isolates EN21 and EN4 demonstrated medium and high solubilization efficiency, respectively. The solubilization index of EN4 and EN21 was 4.0 and 2.2, respectively. Quantitative estimation of solubilized phosphate by potent bacterial isolates, EN4 and EN21, was done by PVK broth method. The amount of solubilized phosphate by the isolates EN4 and EN21 was 376.0 and 173.3 mg/l, respectively (Table 3, Fig. S6).

For zinc solubilization, the results showed that only nine isolates out of 15 isolates could form clearing zones in plate assay. Zinc solubilization potential varied among bacterial isolates (Table 3). The isolate EN4 showed the highest potential of zinc solubilization both in zinc oxide and zinc carbonate-containing media. It produced a clear zone of 16.7 and 15.7 mm with solubilization index of 3.4 and 3.2 in plates containing zinc oxide and zinc carbonate, respectively (Table 3, Fig. S7). HCN production by the bacterial isolates was observed as a change in color of the filter paper from yellow to orange brown. None of the tested isolates was found positive to HCN production (Table 2, Fig. S8).

### Molecular Identification of the Bacterial Isolates

**Fig. 5:**
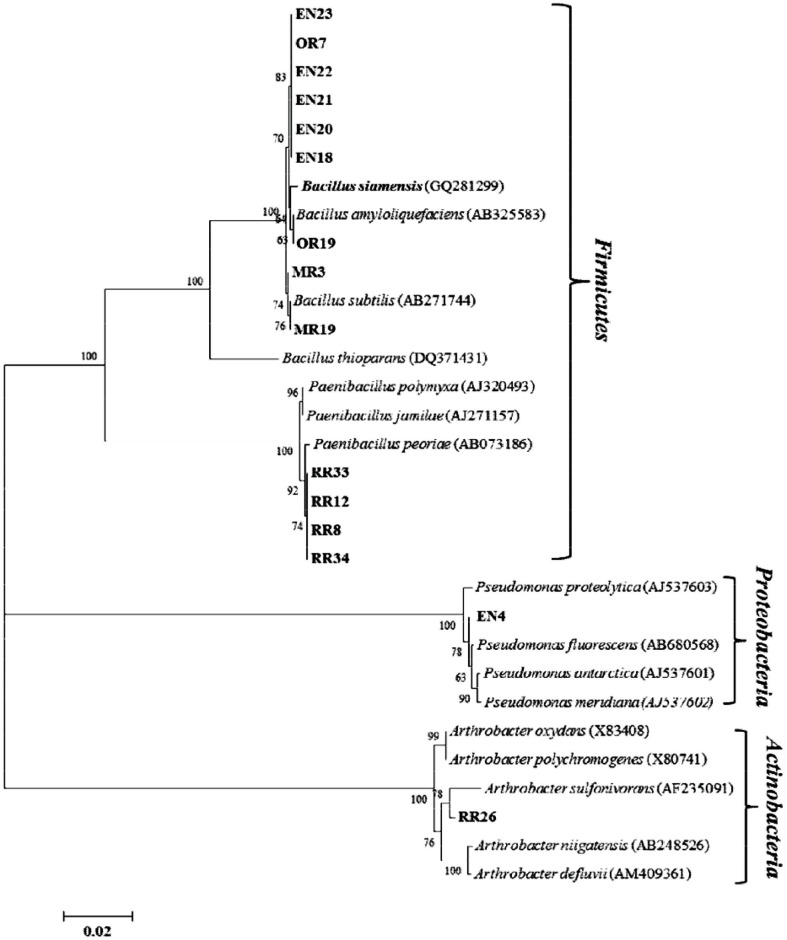
Phylogenetic analysis of internal transcribed spacer regions (16S rRNA gene sequences) of rhizobacteria isolated from various places in Gangwon-do, Korea. MEGA 6 software was used to construct the phylogenetic tree. Boldface indicates the sequences obtained in this study. Numerical values (>50) on branches indicates the percentage of 1,000 bootstrap replicates that support the branch. The scale bar expressed the number of changes per site.

The molecular analysis revealed that 15 isolates belonged to three groups, Firmicutes, Proteobacteria, and Actinobacteria (Fig. 5). Most of the antagonistic bacteria (13 isolates, 86.6% of total) belonged to the Firmicutes group. Phylogenetic analysis based on 16S rRNA gene sequences indicated that *Bacillus* isolates were closely related to the species *Bacillus subtilis* (2 isolates), *Bacillus amyloliquefaciens* (1 isolate) and *Bacillus siamensis* (6 isolates) with the sequence similarities of 99.7-100.0%, 99.8% and 99.4-99.5%, respectively. The four remaining Firmicutes were assigned to *Paenibacillus peoriae* with similarity of 99.0-99.6%. Two isolates, RR26 and EN4, were assigned to *Arthrobacter sulfonivorans* and *Pseudomonas proteolytica* based on their similarities of 98.6% and 99.0, respectively (Fig. 5). Accession numbers for all the identified bacteria isolates were presented in Table 4.

### Suppression of *F. oxysporum* f. sp. *lactuace* and Growth Promotion on Lettuce under In Vitro and In Vivo Conditions

**Fig. 6:**
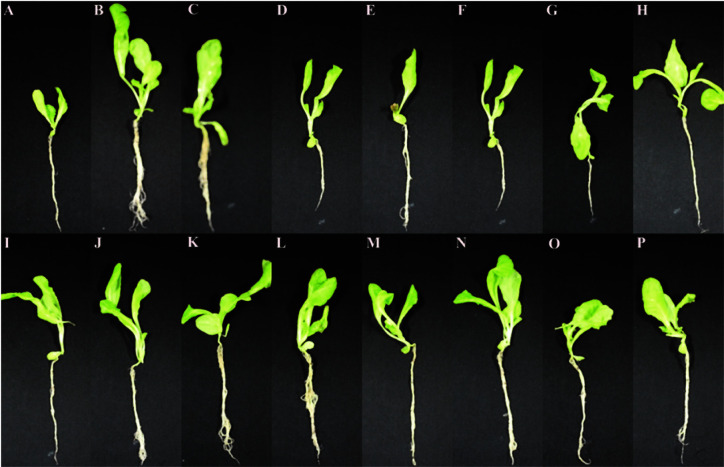
Efficacy of bacterial isolates on lettuce seedling growth by test tube method. (**A**) Negative control; (**B**) Positive control; (**C**) RR8; (**D**) RR12; (**E**) RR26; (**F**) RR33; (**G**) MR3; (**H**) MR19; (**I**) OR7; (**J**) OR19; (**K**) EN4; (**L**) EN18; (**M**) EN20; (**N**) EN21; (**O**) EN22, and (**P**) EN23.

**Fig. 7:**
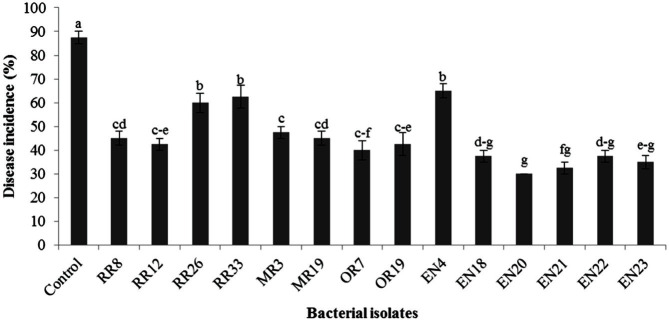
Disease occurrence caused by *Fusarium oxysporum* f. sp. *lactucae* on radicles of lettuce seeds (cv. *Jukchima*) treated with bacterial strains. Germinated lettuce seeds treated with distilled water (control) or bacterial suspensions for 2 h were placed on to the margin of actively growing mycelia of *Fusarium oxysporum* f. sp. *lactucae* on water agar containing 0.02% glucose for 7 days. Lowercase letters expressed the significant differences at *p* ≤ 0.05. The experiment was conducted with four replications of 5 seeds each. Square root transformed data were used for data analysis.

The growth of lettuce seedlings with and without bacterial inoculation, based on root and shoot length and dry weight of whole plant, after 14 days of treatments, is presented in Table 5. The seed inoculations with bacterial strains increased the mentioned growth parameters over negative control and the increment was significant (*p* ≤ 0.05) for most of the isolates. The highest values in all growth parameters were recorded in uninoculated positive control (chemical fertilizer) followed by isolates EN4 and EN21. Visual observation indicated that seedling growth in these two isolates was slightly poor as compared to uninoculated positive control (Fig. 6 and Table 5). Among the tested isolates, isolate EN4 showed increased root length, shoot length and dry weight of whole plant by 96.7, 60.6 and 142.7%, respectively over negative control. In addition, disease incidence was observed highest in control as compared to ones treated with bacterial strains (Fig. 7).

**Fig. 8:**
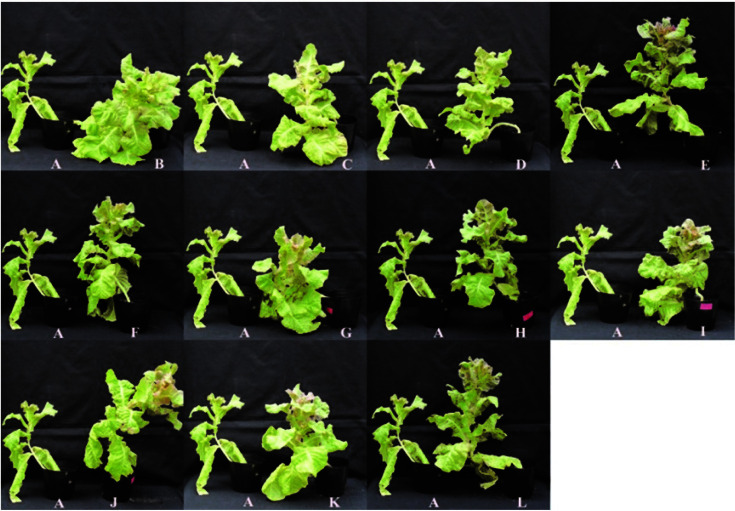
Shoot growth promotion on lettuce by bacterial isolates under greenhouse conditions. (**A**) Negative control; (**B**) Positive control; (**C**) RR8; (**D**) MR19; (**E**) OR7; (**F**) OR19; (**G**) EN4; (**H**) EN18; (**I**) EN20; (**J**) EN21; (**K**) EN22; and (**L**) EN23.

The results of the greenhouse experiment revealed that inoculation with bacterial isolates significantly promoted the growth of lettuce plants over negative control. However, the rate of enhancement varied with bacterial strains. Of tested isolates, isolate EN4 extensively increased all the growth attributes by recording 44.80 cm plant height, 1428.67 cm^2^ leaf area per plant, 38.40 chlorophyll content SPAD value, 1.80 g of root dry weight per plant, 6.35 g of shoot dry weight per plant and 20.50 cm root length (Table 6 and Fig. 8). The results were significantly higher than negative control and most of the bacterial isolates. The results revealed that the effects of isolates EN4 and EN21 were comparable to chemical fertilizer though all the crop attributes were significantly higher in plants treated with chemical fertilizer. Moreover, EN21 showed highest suppression (66.11%) of tested pathogen under greenhouse conditions (Table 7). The results also showed that plants inoculated with any of the tested bacterial isolates significantly reduced wilting percentage (Fig. 7). The highest disease severity reduction was observed with isolate EN21 and then by EN23. The reductions in disease severity by these two isolates were 66.11 and 60.68%, respectively (Table 7). The lowest reductions were produced by isolates EN4 and RR8 (26.21 and 32.06%, respectively). The isolate EN21 caused a 140.5% increment in dry shoot weight over infected control by reducing wilting (Table 7).

**Fig. 9:**
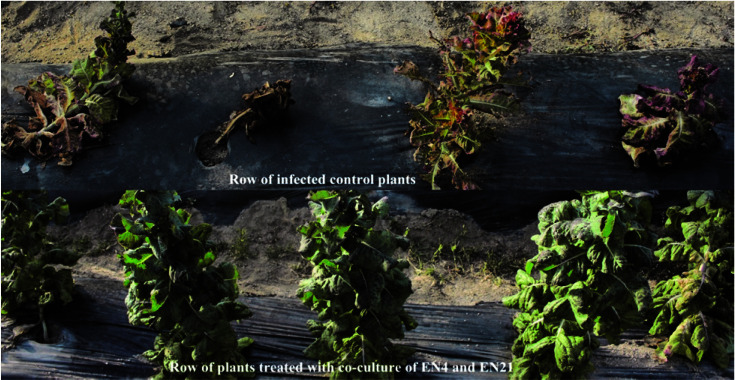
Effect of inoculation with rhizobacteria on development of Fusarium wilt and foliage yield of lettuce under field conditions.

Inoculation of plants with *F. oxysporum* f. sp. *lactucae* caused a significant reduction in shoot dry weight as compared to uninoculated plants under field conditions. Results presented in and Fig. 9 and Table 8 showed that plants inoculated with isolate EN21and a combination of isolates EN4 and E21 significantly reduced wilting percentage in lettuce plants. The highest disease reduction (63.39%) was observed in plants treated with chemical but it was on a par with the disease reduction (57.15%) caused by the combination of isolates EN21 and EN4. The reduction in disease severity by the isolate EN21 was 44.91% when applied in isolation. The use of isolate EN21 with isolate EN4 exhibited significant increase in shoot length over chemical and non-infected control (Table 8 and Fig. 9).

## Discussion

Soil microorganisms are regarded as an important and essential component of soil quality due to their crucial activities in many ecosystem processes [35, 36]. Rhizospheres have been frequently exploited as an excellent source of biocontrol agents, since they provide the frontline of defensive microorganisms for roots against the attack of soil-borne pathogens [37]. In this study, 20 antagonistic bacterial isolates out of 118 rhizobacterial isolates were screened with 13 fungal pathogens as targets. The antagonistic bacterial isolates exerted varied levels of antagonism against tested pathogens. Fluctuation in the spectrum of antifungal activity of bacteria is common [38]. In dual culture assays, isolates RR8, MR3, MR19, OR7, OR19, EN18, EN20, EN21, EN22, and EN23 showed maximum inhibition of radial growth of test pathogens. In this study, some bacterial isolates were found to be highly inhibitory of fungal growth whereas others showed only minor activity or no activity at all. The inhibition zone exhibited between the fungal pathogens and bacteria was expressed in the inhibition of fungal mycelium. Moreover, as the PDA medium used for the dual culture assay is rich in nutrients, competition might be excluded as the mode of action for these isolates [39]. The antifungal metabolites produced seems to vary among the bacterial isolates tested in this study. This suggests that the fungal mycelia might not only be inhibited by antibiosis but also by other antifungal metabolites such as siderophores, hydrogen ions and gaseous products including ethylene, hydrogen cyanide and ammonia [40]. In vitro broth-based dual cultures offer a better method for evaluation of antagonistic efficiency of the biocontrol agents as the liquid medium may provide a better environment to allow the antagonistic activities from all possible interacting sites. These results are in agreement with the findings of Ashwini and Srividya (2014) [41] who revealed that antagonistic bacteria, *Bacillus subtilis*, inhibited *C. gloeosporioides* up to 100% in terms of dry weight and caused a clear hyphal lysis and degradation of fungal cell wall.

This study revealed that some rhizobacterial isolates were capable of inhibiting a wide range of phytopathogens in controlled conditions. But, in most biocontrol investigations, a large number of antagonists are commonly isolated over a short period of time and screened in vitro for antagonistic activity and tests based on in vitro mycelial inhibition and root colonization do not always correlate with biocontrol efficacy under natural conditions [42]. However, little correlation exists between in vitro and in vivo antagonistic activity in general [43] and identification of promising field-effective bacteria, however, can be facilitated by greenhouse experiments [44]. The major bacterial genus identified in our studies was *Bacillus* and these bacteria were also found in the rhizosphere of crop plants [45].

In the present study, *Pseudomonas proteolytica* (EN4) and *Bacillus* spp. (MR3, MR19, OR7, OR19, EN18, EN20, EN21, and EN23) were found positive for most of the antifungal traits. Our results indicated that stress conditions favor siderophore production. None of our isolates were positive to HCN and it might be due to the fact that cyanogenesis is minimal during bacterial transition from exponential to stationary phases of growth change and cyanogen production is dependent on environmental factors such as iron and phosphorus availability [46]. Most of our studied isolates were chitinase producers, forming halos of clearance on chitin media. Hence, we screened 118 bacteria for an in vitro evaluation of antifungal activities in order to select the ones that show potential as well. The results revealed that some of the antagonistic bacteria exhibited antifungal traits under in vitro conditions. Such multiple modes of action have been reported to be the main reasons for the plant growth promotion and disease-suppressing efficacy of bacteria [47]. Wilt caused by different forma species of *F. oxysporum* is a disastrous disease of lettuce. Conventional control of disease depends on the use of chemical inputs and resistant varieties. Development of new variants of the fungus, health hazards, and environmental pollution concerned with the excessive use of agro-chemicals have resulted in adopting biological control using native strains of plant-associated rhizobacteria as a supplemental strategy to minimize pesticide usage [48]. In comparison with negative control, the best results were demonstrated by *B. siamensis* EN21 and *P. proteolytica* EN4 on all tested plants (lettuce). The difference in the response of tested isolates between in vitro and in vivo conditions might be attributed to the change in overall environmental condition that favors disease development. A strain of *P. fluorescens* inoculated near the roots of carnation also protected the plants against Fusarium wilt by suppressing and resisting the stem-inoculated pathogen *F. oxysporum* f. sp. *dianthi* [49]. Rhizobacteria has opened new horizons and facilitated the the design different strategies by researchers to get maximum benefit from the tiny creature and improve the efficacy of biocontrol agents [50]. In light of this, the focus of the work is directed towards isolating and identifying the antagonistic rhizobacteria possessing plant growth-promoting ability both in in vitro and in vivo conditions in lettuce crop. Further studies regarding the detail inside the mechanisms of rhizobacteria and their investigation at the farm level have been increasing in number in recent days.

## Supplemental Materials

Supplementary data for this paper are available on-line only at http://jmb.or.kr.

## Figures and Tables

**Table 1:** Antagonistic efficacy of rhizobacterial isolates against *F. oxysporum* f. sp. *lactucae* in dual culture assay.

Isolates	Fungal pathogens

	*F. oxysporum* f. sp. *lactucae*
RR8	56.1^d^ (5.0^d-e^)
RR12	52.9^e^ (4.3^d-f^)
RR26	54.5^de^ (4.7^de^)
RR33	49.8^f^ (3.3^f^)
RR34	55.7^d^ (5.3^d^)
MR3	53.7^de^ (5.0^de^)
MR19	62.0^c^ (3.7^ef^)
OR7	66.3^a^ (7.7^c^)
OR19	63.1^bc^ (5.7^d^)
EN4	0.0^g^ (0.0^g^)
EN18	63.9^a-c^ (8.7^bc^)
EN20	65.5^ab^ (8.3^c^)
EN21	66.3^a^ (10.0^a^)
EN22	65.4^ab^ (8.7^bc^)
EN23	65.5^ab^ (9.7^ab^)
Control	0.0^g^ (0.0^g^)

**Table 2:** Antagonistic traits of selected antagonistic bacterial isolates.

Isolates	Hydrolytic enzymes				HCN production	Siderophore production	Chitinase	Endozymes	
	
	Protease	Cellulase	Pectinase	α-amylase				Catalase	Oxidase
RR8	++	+++	++	++	-	-	+++	++	++
RR12	++	+++	++	++	-	+	+++	++	++
RR26	+++	+++	+++	+++	-	+	+++	+++	+++
RR33	+	+++	++	++	-	+	+	+	+
RR34	-	+	-	-	-	+	+++	+	+
MR3	++	+++	++	++	-	+++	+++	++	++
MR19	++	+++	++	++	-	+++	+++	+++	+++
OR7	+++	+++	+++	+++	-	+++	+++	+++	+++
OR19	+++	+++	+++	+++	-	+++	+++	+++	+++
EN4	+++	-	-	-	-	+++	+	++	++
EN18	+++	+++	+++	+++	-	+++	+++	++	++
EN20	+++	+++	+++	+++	-	+++	+++	++	++
EN21	+++	+++	+++	+++	-	+++	+++	++	++
EN22	+++	+++	+++	+++	-	+++	+++	+	++
EN23	+++	+++	+++	+++	-	+++	+++	++	++

+++ = high, ++ = medium, + = low, - = negative producer.

**Table 3:** Growth promoting traits of selected antagonistic bacterial isolates.

Isolates	IAA^†^	NH_3_ production^†^	Phosphate solubilization^α^	Zinc solubilization^α^
RR8	-	-	+	+
RR12	-	-	+	+
RR26	-	++	-	-
RR33	-	-	-	+
RR34	-	+	-	++
MR3	-	++	-	+++
MR19	-	++	-	+
OR7	+	++	-	-
OR19	-	++	-	++
EN4	++	+++	+++	+++
EN18	-	++	-	-
EN20	-	++	-	-
EN21	++	+++	++	-
EN22	-	++	-	-
EN23	-	++	+	+++

^†^+++ = strong, ++ = medium, + = weak and - = no production of IAA and NH_3_;

^α^+++ = strong, ++ = medium, + = weak and - = no solubilization of phosphate and zinc.

**Table 4:** Similarity scores between bacterial isolates and the highly matched type strain identified by neighbor-joining analysis.

Bacterial isolates	Closest GenBank accession No.	Closest GenBank taxa	Similarity (%)
RR8 (KU512890)	AB073186	*Paenibacillus peoriae*	99.5
RR12 (KU512891)	AB073186	*Paenibacillus peoriae*	99.0
RR26 (KU512892)	AF235091	*Arthrobacter sulfonivorans*	98.6
RR33 (KU512893)	AB073186	*Paenibacillus peoriae*	99.1
RR34 (KU512894)	AB073186	*Paenibacillus peoriae*	99.1
MR3 (KU512895)	AB271744	*Bacillus subtilis*	99.7
MR19 (KU512896)	AB271744	*Bacillus subtilis*	100.0
OR7 (KU512897)	GQ281299	*Bacillus siamensis*	99.5
OR19 (KU512898)	AB325583	*Bacillus amyloliquefaciens*	99.8
EN4 (KU512899)	AJ537603	*Pseudomonas proteolytica*	99.0
EN18 (KU512900)	GQ281299	*Bacillus siamensis*	99.5
EN20 (KU5129101)	GQ281299	*Bacillus siamensis*	99.4
EN21 (KU5129102)	GQ281299	*Bacillus siamensis*	99.4
EN22 (KU5129103)	GQ281299	*Bacillus siamensis*	99.4
EN23 (KU5129104)	GQ281299	*Bacillus siamensis*	99.4

**Table 5:** Efficacy of bacterial isolates on lettuce seedling growth by test tube method in vitro.

Isolates	Shoot length (cm)	Root length (cm)	Seedling weight (mg/seedling)	

			Fresh	Dry
RR8	10.57^d^	11.60^ef^	775.77^g^	45.03^d^
RR12	10.43^d^	10.80^fg^	583.03^k^	40.10^d^
RR26	9.47^e^	9.80^h^	552.70^l^	32.02^e^
RR33	7.40^f^	10.07^gh^	549.87^l^	30.69^e^
MR3	9.43^e^	11.00^f^	608.35^j^	43.36^d^
MR19	9.60^e^	10.90^f^	630.43^i^	43.03^d^
OR7	10.63^d^	12.47^c-e^	761.90^g^	45.20^d^
OR19	10.53^d^	12.43^c-e^	707.68^h^	43.03^d^
EN4	12.53^b^	13.33^b^	1235.90^b^	74.50^a^
EN18	10.67^d^	12.33^c-e^	873.53^f^	55.13^c^
EN20	10.97^d^	12.27^c-e^	1035.68^e^	54.23^c^
EN21	12.20^bc^	13.03^bc^	1164.36^c^	65.35^b^
EN22	11.67^c^	12.03^de^	882.70^f^	45.36^d^
EN23	11.67^c^	12.70^b-d^	1128.36^d^	66.37^b^
Positive Control	13.20^a^	15.07^a^	1321.83^a^	75.34^a^
Negative Control	6.37^g^	8.30^i^	533.17^m^	30.70^f^

Data are means of 10 replications.

Values with different alphabetic superscripts in the same column are significantly different at p ≤ 0.05 levels according to Duncan’s multiple range test.

**Table 6:** Effects of bacterial isolates on growth parameters of lettuce in soil treatments under greenhouse conditions.

Isolates	Plant height (cm)	Leaf area (cm^2^/plant)	Chlorophyll content (SPAD value)	Fresh weight (g/plant)		Dry weight (g/plant)		Root length (cm)

				Root	Shoot	Root	Shoot	
RR8	34.17^fg^	1235.67^h^	31.03^gh^	8.47^f^	51.98^g^	0.85^g^	4.12^ef^	15.53^g^
MR19	34.13^fg^	1230.33^h^	30.77^h^	8.44^f^	51.90^g^	0.82^g^	3.71^f^	15.53^g^
OR7	35.57^e^	1330.00^g^	32.80^ef^	12.71^e^	53.48^g^	1.14^e^	4.69^d^	16.33^f^
OR19	35.03^ef^	1327.00^g^	31.87^fg^	12.39^e^	53.22^g^	0.96^f^	4.58^de^	16.13^fg^
EN4	44.80^b^	1428.67^b^	38.40^b^	18.73^b^	92.29^b^	1.80^b^	6.35^a^	20.50^b^
EN18	35.47^e^	1346.33^f^	34.83^d^	13.52^d^	71.02^d^	1.29^d^	4.97^cd^	17.33^de^
EN20	33.50^g^	1320.67^g^	33.47^e^	13.45^d^	62.01^f^	1.28^d^	4.71^d^	16.63^ef^
EN21	42.67^c^	1415.67^c^	36.93^c^	18.51^b^	75.94^c^	1.72^b^	5.56^b^	18.50^c^
EN22	35.87^e^	1362.67^e^	35.57^d^	14.64^c^	67.79^e^	1.50^c^	5.03^cd^	17.43^d^
EN23	38.53^d^	1391.67^d^	35.13^d^	14.75^c^	71.32^d^	1.53^c^	5.37^bc^	18.30^c^
Positive Control	51.63^a^	1530.00^a^	40.70^a^	20.70^a^	98.37^a^	2.87^a^	6.72^a^	21.47^a^
Negative Control	31.17^h^	462.33^i^	28.50^i^	7.51^g^	41.43^h^	0.75^h^	2.85^g^	13.20^h^

Data are means of five replications.

Values with different alphabetic superscripts in the same column are significantly different at p ≤ 0.05 levels according to Duncan’s multiple range test.

**Table 7:** Effect of inoculation with rhizobacteria on development of Fusarium wilt and shoot dry weight on lettuce under greenhouse conditions.

Treatments^a^	Disease severity^b^ (%)	Disease reduction (%)	Shoot dry weight (g/plant)
RR8	65.67^bc^	32.06	3.67^g^
MR19	61.33^c^	36.56	3.67^g^
OR7	45.33^d^	53.10	4.45^e^
OR19	44.00^de^	54.47	4.47^e^
EN4	71.33^b^	26.21	4.2^f^
EN18	42.00^de^	56.54	4.85^c^
EN20	44.00^de^	54.49	4.72^d^
EN21	34.67^fg^	66.11	6.35^a^
EN22	40.33d^ef^	58.27	4.93^c^
EN23	38.00^e-g^	60.68	6.12^b^
Chemical	32.67^g^	64.12	2.92^h^
Non-infected Control	-	-	2.75^i^
Infected Control	96.67^a^	-	2.64^j^

**Table 8:** Effect of inoculation with rhizobacteria on development of Fusarium wilt and shoot length of lettuce under field conditions.

Treatments^a^	Shoot length (cm)	Disease severity^b^ (%)	Disease reduction (%)
EN21	85.17^b^	45.9^b^	44.91
EN4+21	94.83^a^	35.7^cd^	57.15
Chemical	74.83^c^	30.5^d^	63.39
Non-infected Control	64.67^d^	-	-
Infected Control	28.17^e^	83.33^a^	-

^a^Lettuce plants (cv. Jukchima) were treated by drenching the soil around root zone with the broth culture of bacterial isolates two times at an interval of seven days. Control plants (not infected and infected control) were treated with tap water and plants were sprayed with 0.2% solution of Mancozeb 80WP two times at an interval of seven days.

^b^Disease severity was recorded at 8 weeks after planting.

Data are means of five replications. Values with different alphabetic superscripts in the same column are significantly different at *p* ≤ 0.05 levels according to Duncan’s multiple range test.
