# Supplementary material for: Suppression of Fusarium Wilt Caused by Fusarium oxysporum f. sp. lactucae and Growth Promotion on Lettuce Using Bacterial Isolates
Source: J Microbiol Biotechnol. 2021 Aug 4;31(9):1241–55. doi: 10.4014/jmb.2104.04026 (PMC9705851; doi:10.4014/jmb.2104.04026)

J. Microbiol. Biotechnol.
https://doi.org/10.4014/jmb.2104.04026

Supplementary figure 1

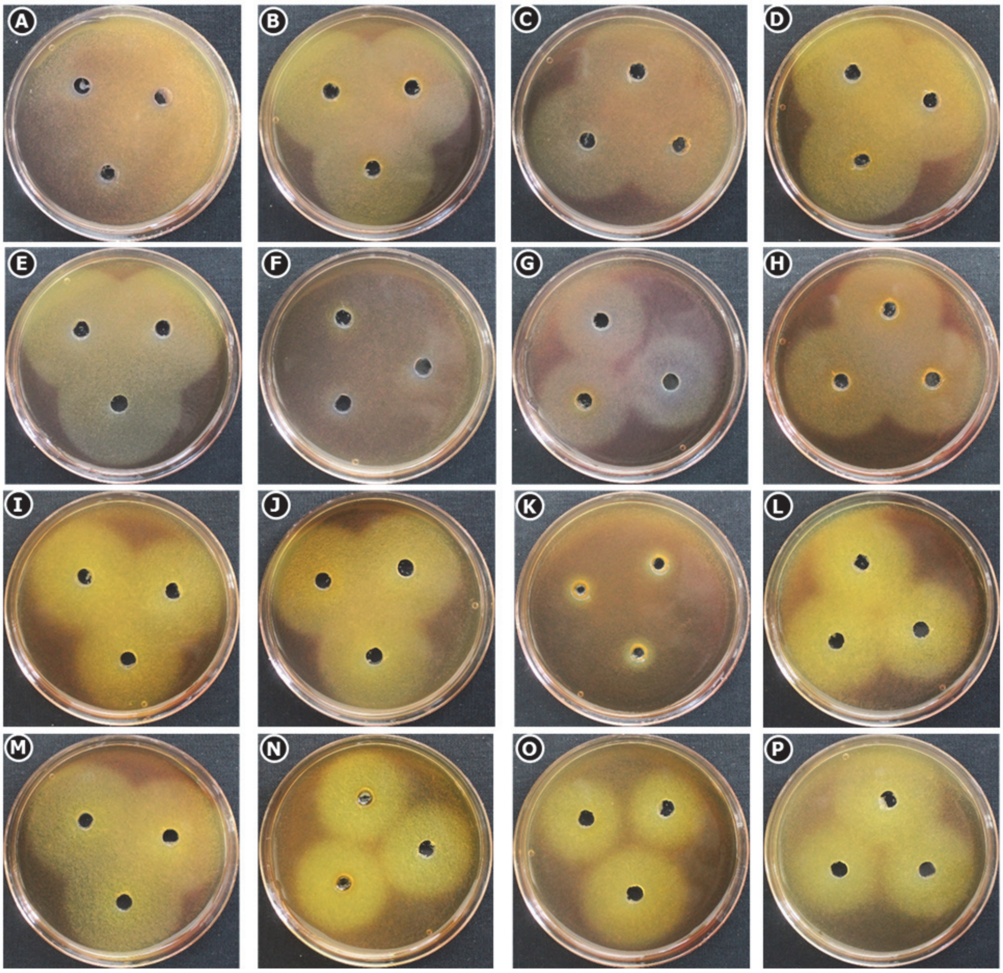

Supplementary figure 2

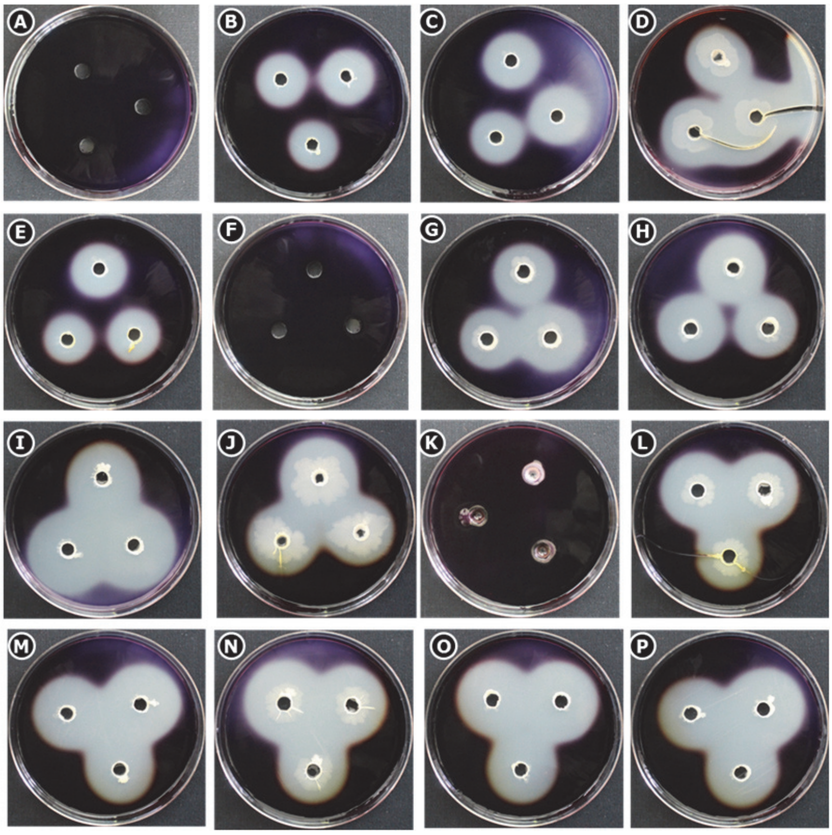

Supplementary figure 3

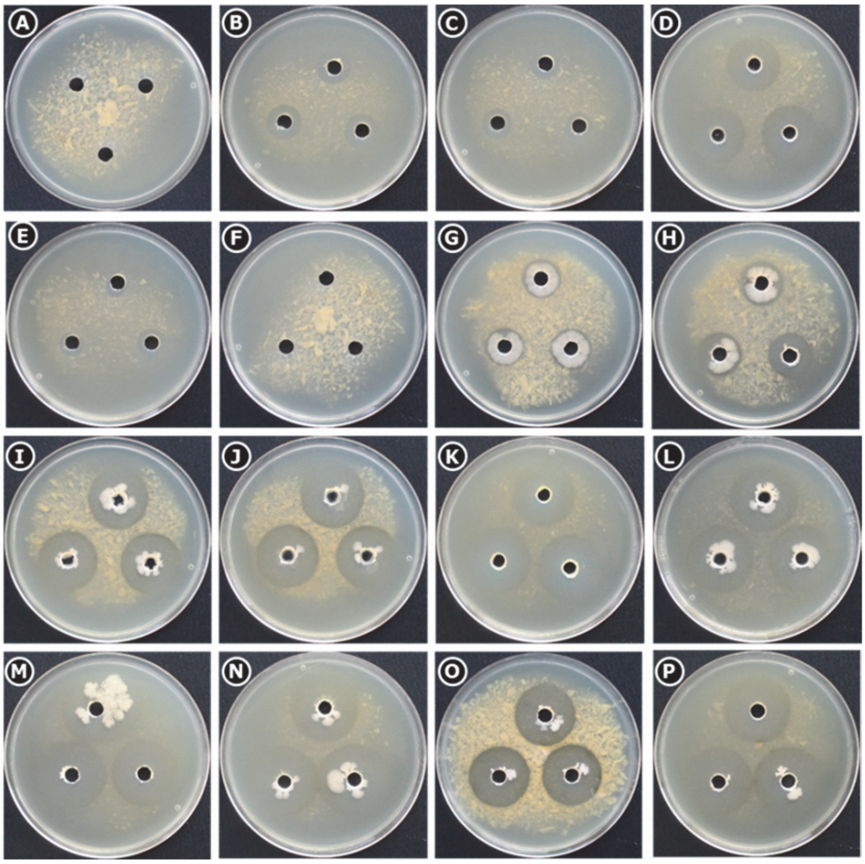

Supplementary figure 4

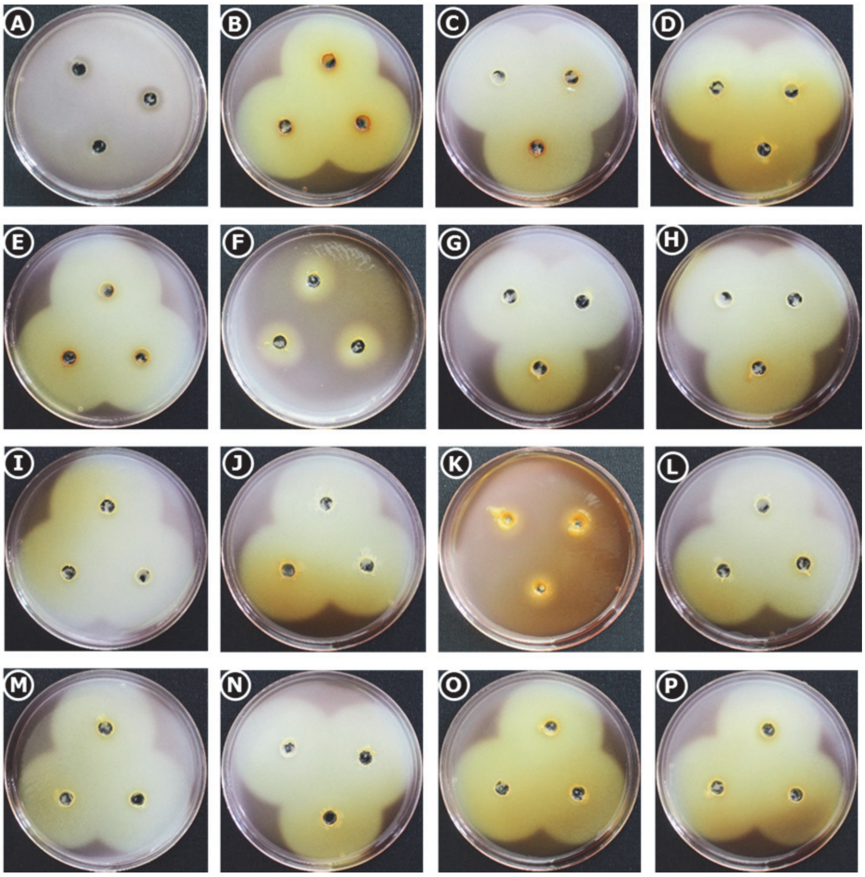

Supplementary figure 5

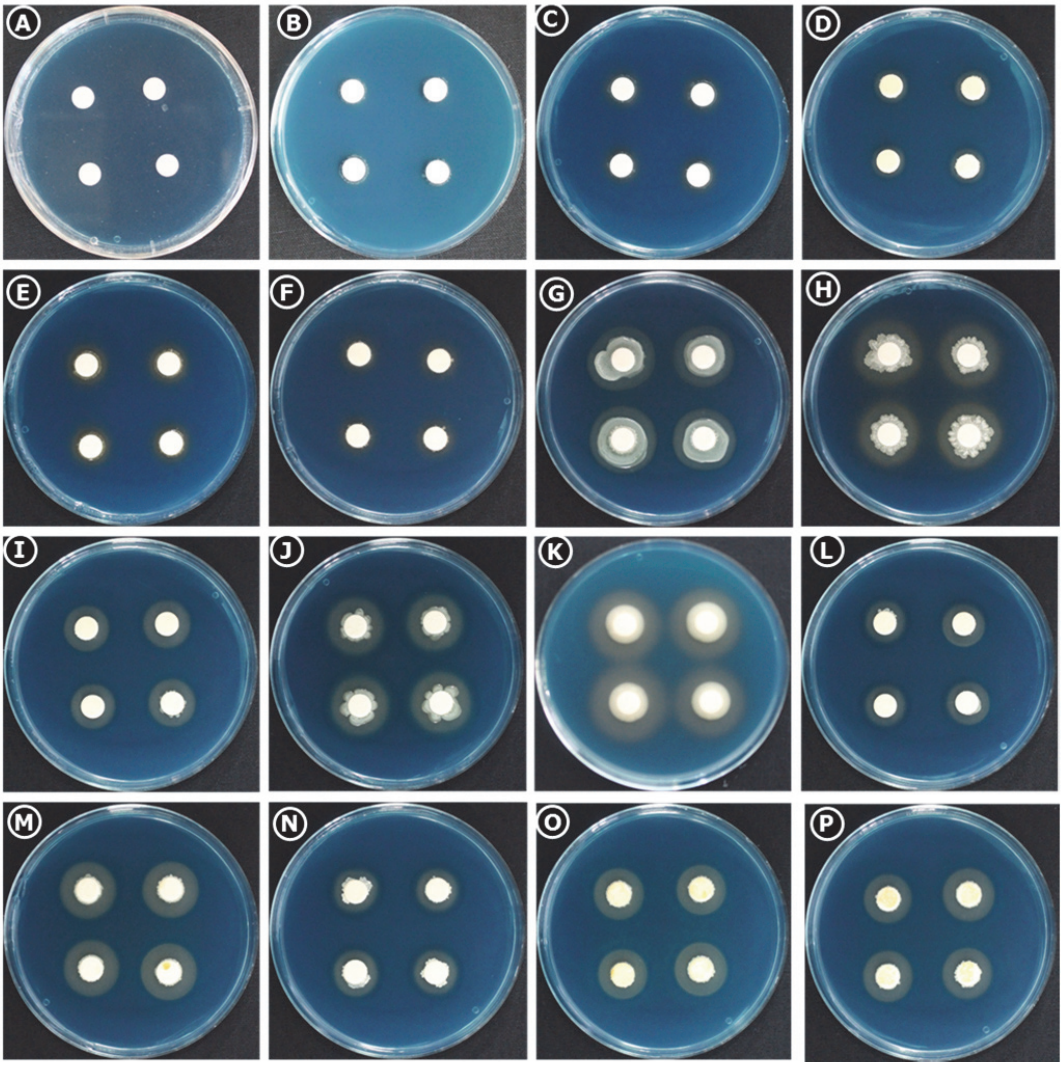

Supplementary figure 6

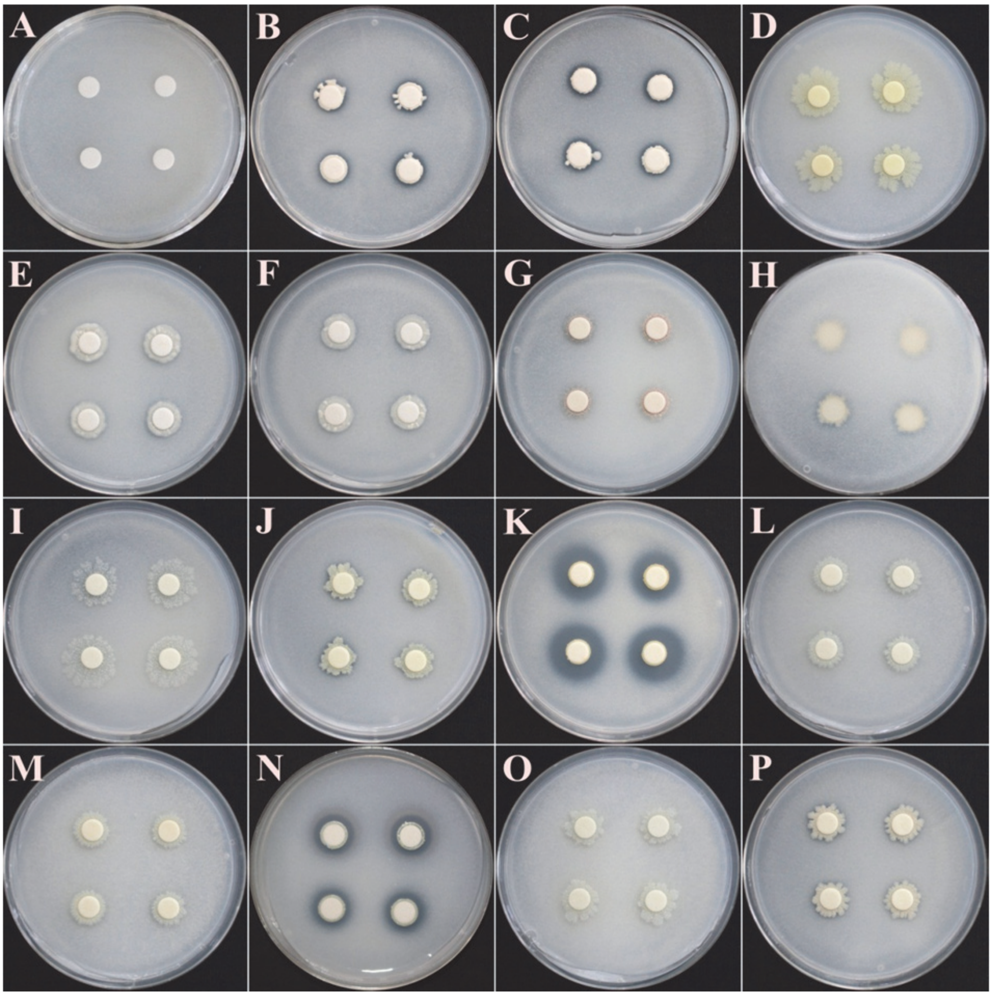

Supplementary figure 7

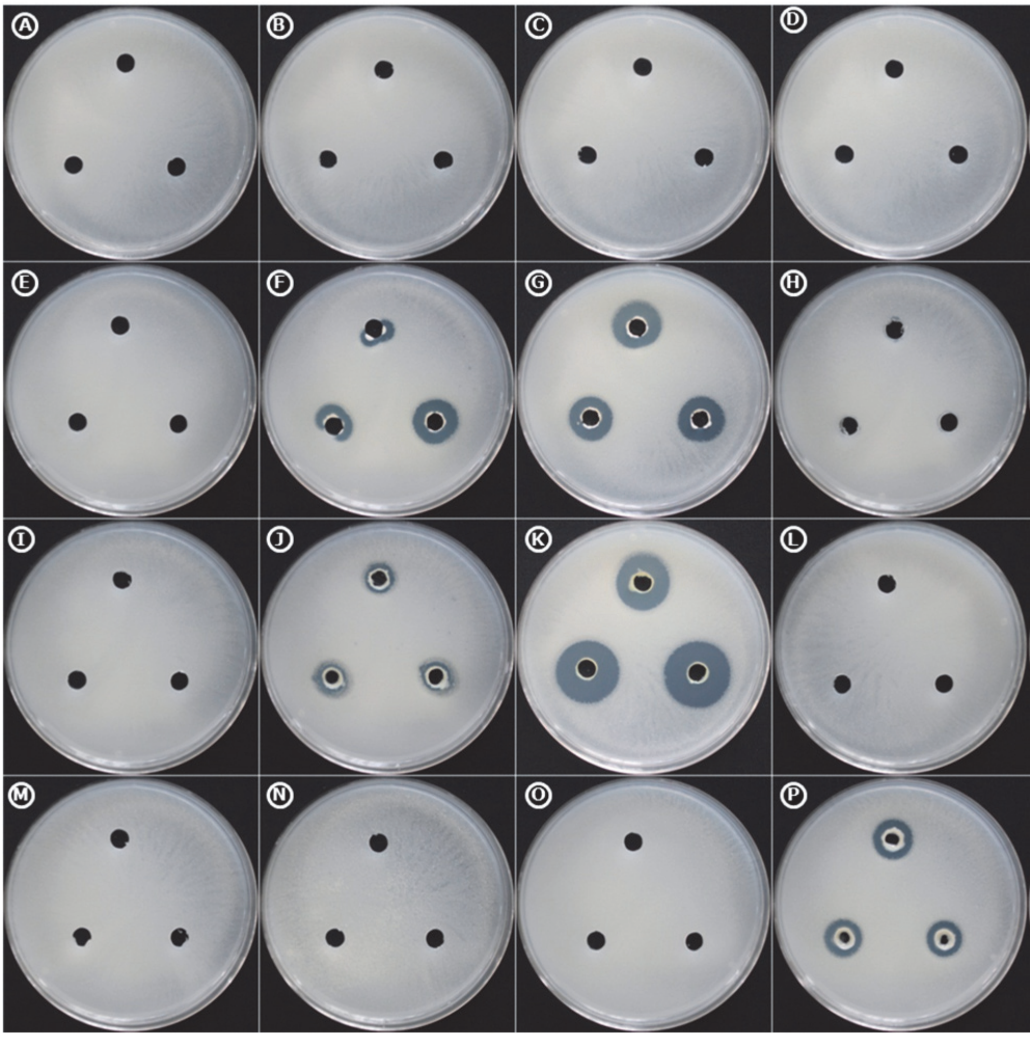

.

Supplementary figure 8

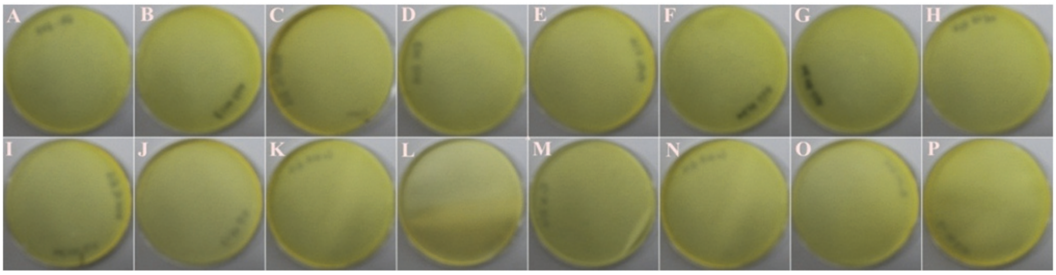

Supplement: Supplementary file 1 [file jmb-31-9-1241-supple.pdf]
